# RTA Promoter Demethylation and Histone Acetylation Regulation of Murine Gammaherpesvirus 68 Reactivation

**DOI:** 10.1371/journal.pone.0004556

**Published:** 2009-02-23

**Authors:** Zhangsheng Yang, Haidong Tang, Hai Huang, Hongyu Deng

**Affiliations:** 1 Center for Infection and Immunity and National Laboratory of Biomacromolecules, Institute of Biophysics, Chinese Academy of Sciences, Beijing, China; 2 Graduate School of the Chinese Academy of Sciences, Beijing, China; 3 School of Dentistry, University of California Los Angeles, Los Angeles, California, United States of America; University of Hong Kong, Hong Kong

## Abstract

Gammaherpesviruses have a common biological characteristic, latency and lytic replication. The balance between these two phases in murine gammaherpesvirus 68 (MHV-68) is controlled by the replication and transcription activator (RTA) gene. In this report, we investigated the effect of DNA demethylation and histone acetylation on MHV-68 replication. We showed that distinctive methylation patterns were associated with MHV-68 at the RTA promoter during latency or lytic replication. Treatment of MHV-68 latently-infected S11E cells with a DNA methyltransferases (DNMTs) inhibitor 5-azacytidine (5-AzaC), only weakly reactivated MHV-68, despite resulted in demethylation of the viral RTA promoter. In contrast, treatment with a histone deacetylase (HDAC) inhibitor trichostatin A (TSA) strongly reactivated MHV-68 from latency, and this was associated with significant change in histone H3 and H4 acetylation levels at the RTA promoter. We further showed that HDAC3 was recruited to the RTA promoter and inhibited RTA transcription during viral latency. However, TSA treatment caused rapid removal of HDAC3 and also induced passive demethylation at the RTA promoter. *In vivo*, we found that the RTA promoter was hypomethylated during lytic infection in the lung and that methylation level increased with virus latent infection in the spleen. Collectively, our data showed that histone acetylation, but not DNA demethylation, is sufficient for effective reactivation of MHV-68 from latency in S11E cells.

## Introduction

Murine gammaherpesvirus 68 (MHV-68, also referred to as γHV68), is a member of the gammaherpesvirus subfamily [1]. MHV-68 is phylogenetically related to two other human gammaherpesviruses, Epstein-Barr Virus (EBV) and Kaposi's sarcoma-associated herpesvirus (KSHV), which are associated with lymphoproliferative diseases and several human tumors [1], [2], [3], [4]. Due to the difficulty in culturing EBV and KSHV *in vitro* and the lack of a good *in vivo* system to directly study them, MHV-68 has been used as an *in vitro* and *in vivo* model for gammaherpesvirus infection [3], [5], [6], [7].

Herpesviruses have two distinct life cycle phases, latency and lytic replication. Latent infection is thought to be important for tumorigenesis associated with these viruses. Reactivation from latency to lytic replication is essential for transmission of the virus from host to host; it has been suggested that even low-frequency viral reactivation plays a significant role in viral pathogenesis [6], [8]. A viral protein, replication and transcription activator (RTA), primarily encoded by open reading frame (ORF) 50, is well conserved among gammaherpesviruses [9], [10], [11], [12]. RTA is regarded as a “molecular switch” controlling reactivation of KSHV and MHV-68; both KSHV RTA and MHV-68 RTA are sufficient and necessary to reactivate their respective viruses from latently infected cells [10], [13], [14], [15].

Chromatin modifications, including DNA methylation and histone acetylation, play an important role in regulating gene transcription [16]. Histone deacetylases (HDACs), which act in opposition to histone acetyltransferases (HATs), control the level of histone acetylation and serve as means for post-translational modification of nucleosomal histones that influence gene expression [17]. Histone acetylation promotes gene transcription by relaxing chromatin structure and facilitating access to DNA by the transcriptional machinery, whereas histone deacetylation promotes transcriptional repression by condensing chromatin structure. It has been reported that KSHV was reactivated from latency after 5-azacytidine (5-AzaC) treatment of primary effusion lymphoma-derived cell lines [18]. In addition, HDAC inhibitors activate KSHV RTA promoter strongly and HDACs are recruited to the RTA promoter [19]. Studies of EBV have also shown that chromatin modifications regulate virus reactivation 20, 21, 22. However, the effect of DNA demethylation and histone acetylation of MHV-68 reactivation have not been characterized yet. Therefore, the aim of this study was to investigate the role of DNA demethylation and histone acetylation in MHV-68 reactivation. We show that histone acetylation is sufficient for MHV-68 reactivation from latency in S11E cells (a B cell line latently infected with MHV-68), and this process is accompanied by demethylation of the RTA promoter.

## Results

### 1. 5-AzaC treatment weakly led to MHV-68 lytic replication in S11E cells

**Figure 1:**
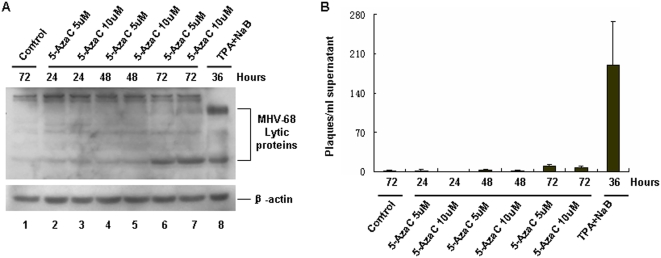
5-AzaC treatment of S11E cells induced weak MHV-68 lytic replication. (A) Detection of MHV-68 lytic protein by western blotting after S11E cells were induced by 5-AzaC. S11E cells were treated with 5 µM or 10 µM 5-AzaC for 24, 48 or 72 hrs, then cells were lysed and viral lytic proteins detected using a polyclonal antibody against MHV-68 lytic antigens. TPA (25 ng/ml) plus NaB (4 mM) treatment served as a positive control. (B) Supernatants from induced S11E cells were collected, and plaque assay were performed to detect viral titers. The experiments were repeated three times and standard deviations were expressed as error bars.

It has been reported that EBV and KSHV can be reactivated from latency by DNA methylation inhibitor reagents such as 5-AzaC [18], [22]. In order to determine if latent MHV-68 virus can also be reactivated by this drug, we treated a MHV-68 latently-infected cell line, namely S11E, with 5-AzaC. 12-O-tetradecanoylphorbol-13-acetate (TPA) plus sodium butyrate (NaB) treatment, which has been reported to activate MHV-68 lytic replication [23], [24], served as a positive control. At indicated time points, cellular extracts were analyzed by western blotting using a polyclonal antibody against MHV-68 lytic antigens. As shown in Fig. 1A, TPA plus NaB treatment for 36 hrs induced MHV-68 reactivation, leading to production of lytic antigens (lane 8), compared to untreated control cells (lane 1). In contrast, treatment of S11E cells with 5 µM or 10 µM 5-AzaC for 24 or 48 hrs, induced little reactivation of MHV-68 virus (lanes 2 to 5). Extending the treatment to 72 hrs resulted in modestly increased level of reactivation (lanes 6 and 7). The different patterns of lytic proteins induced by 5-AzaC vs TPA plus NaB might be attributed to different signaling pathways involved in viral reactivation. To analyze the reactivation efficiency of 5-AzaC more quantitatively, supernatants were collected for plaque assay. As shown in Fig. 1B, treating S11E cells with 5-AzaC for 72 hrs resulted in detectable production of infectious viral particles, compared to untreated control cells. However, this production level is very low, as TPA plus NaB treatment generated a significantly higher number of viral particles compared to untreated control. Taken together, these data indicated that 5-AzaC treatment only led to weak reactivation of MHV-68 from latency.

### 2. RTA promoter methylation was associated with MHV-68 latency *in vitro*

**Figure 2:**
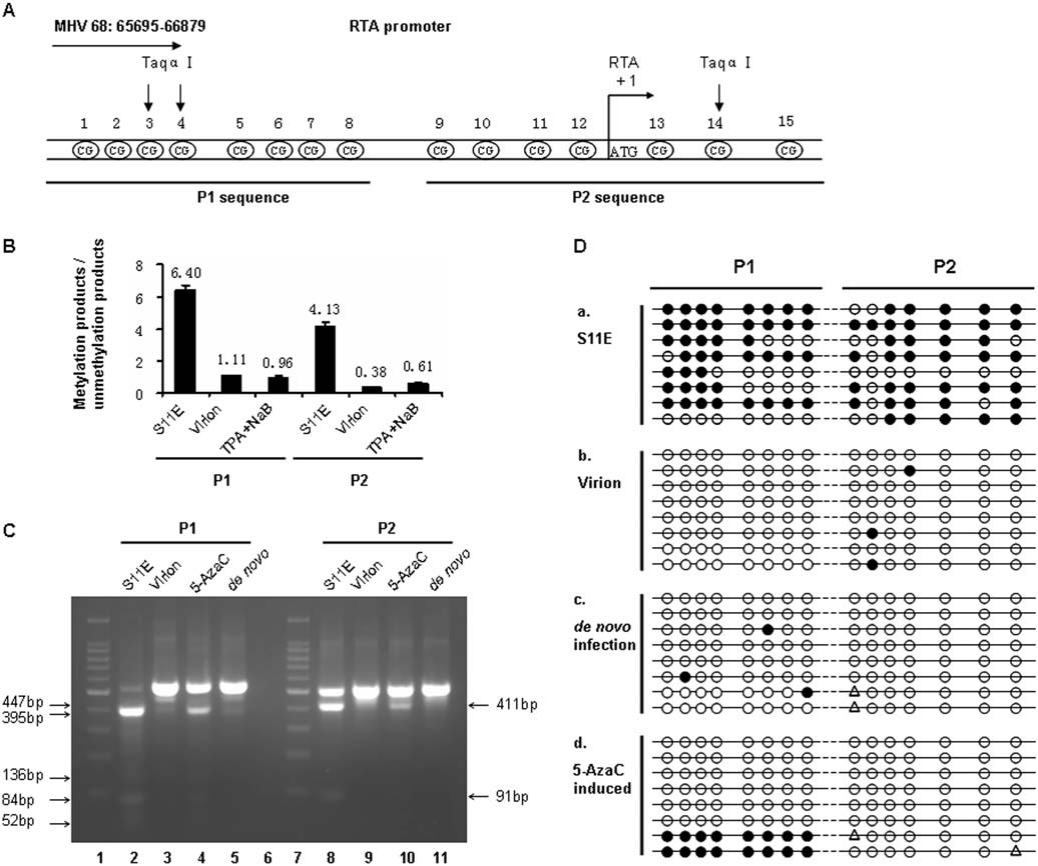
Methylation status at the MHV-68 RTA promoter. (A) A schematic view of the RTA promoter on MHV-68 genome (NC_001826, n.t. 65695-66879). The 15 CpG sites shown were analyzed in this study, the promoter region divided into two parts for analysis, named P1 and P2. (B) Quantitative methylation-specific PCR (Q-MSP) analysis of the RTA promoter methylation status. Genomic DNAs were prepared from untreated S11E cells, MHV-68 virion or TPA (25 ng/ml) plus NaB (4 mM) treated S11E cells. Then Q-MSP was performed to detect methylation status of the RTA promoter P1 and P2 fragments, using methylation-specific (M) or unmethylation-specific (U) primers, respectively. Standard reactions were performed at the same time to quantify copy numbers, and the ratio of methylation-specific and unmethylation-specific products was calculated for each sample. Results represent the average from three experiments, with standard deviations shown. (C) Combined bisulfite restriction analysis (COBRA) of the MHV-68 RTA promoter methylation status. Genomic DNAs were prepared from untreated S11E cells, MHV-68 virion, 5-AzaC (10 µM, 36 hrs) induced S11E cells or *de novo* infected MHV-68 BHK-21 cells, bisulfite modified, and subsequently used for PCR amplification. The PCR products were digested by Taqα| for COBRA. The digested products for P1 fragments are 84 bp, 52 bp, 136 bp, 447 bp and 395 bp. The digested products for P2 fragments are 411 bp and 91 bp. (D) Bisulfite genomic sequencing (BGS) analysis of the 15 CpG sites at the RTA promoter. Genomic DNAs from untreated S11E cells, MHV-68 virion, *de novo* infected MHV-68 BHK-21 cells or 5-AzaC (10 µM, 24 hrs) induced S11E cells, bisulfite modified, and then used for PCR amplification, The PCR products were cloned into T-A vector. Each group included eight independent clones. Solid circles indicate methylated CpG, open circles indicate unmethylated CpG and triangle indicate ambiguous sequencing results.

There were two obvious explanations for the 5-AzaC experiment results: 5-AzaC treatment was unable to induce demethylation of latent MHV-68 genome, or demethylation of the latent MHV-68 genome was not sufficient to induce efficient MHV-68 reactivation. To facilitate examination of these possibilities, we decided to focus on the RTA promoter region on viral genome, since RTA has been shown to be the “molecular switch” for controlling MHV-68 reactivation. Although it has been reported that the whole MHV-68 genome was highly CpG suppressed [18], there are a few CpG sites at the RTA promoter near the ATG initiation cordon for RTA protein synthesis. We thus examined an approximately 1 kb fragment in the proximal RTA promoter region which includes 15 CpG sites. For convenience of technical analysis, we divided this 1 kb fragment into two parts, P1 and P2 (Fig. 2A). We performed quantitative methylation-specific PCR (Q-MSP) to detect methylation status at the RTA promoter in S11E cells during latency and after reactivation. As a comparison, we also analyzed the RTA promoter on viral genome from virions. As shown in Fig. 2B, the ratio of methylation-specific products and unmethylation-specific products was different among these 3 groups of samples, suggesting a much higher percentage of methylated CpG sites on RTA promoter in S11E cells than in virions, and that the percentage of methylated CpG sites dramatically decreased after viral reactivation induced by TPA plus NaB treatment.

To confirm this result, we performed combined bisulfite restriction analysis (COBRA) experiments (Fig. 2C). In this method, sodium bisulfite treatment of DNA fragment converts unmethylated cytosines to uracils. PCR amplification of the converted fragment leads to creation of new, or loss of existing, restriction site (s), and susceptibility or resistance to selected restriction enzyme digestion reveals the methylation status of the original DNA fragment. For the RTA promoter P1 fragment, the majority of the DNA extracted from S11E cells (80–90%) could be digested by Taqα|, indicating methylation of the DNA fragment (Fig. 2C, lane 2). 5-AzaC treatment resulted in a large proportion of the CpG sites (approximately 80%) resistant to Taqα| digestion, indicating demethylation induced by 5-AzaC (Fig. 2C, lane 4). As a comparison, only 10 to 20 percent of the DNA sample from virions could be digested by Taqα|, suggesting that they were mostly in unmethylated status (Fig. 2C, lane 3). The RTA promoter fragment from *de novo* infected BHK-21 cells, were nearly all unmethylated (Fig. 2C, lane 5). Results from the methylation analysis of P2 fragment were similar to those of the P1 part (Fig. 2C, lanes 8–11).

Both MSP and COBRA analyses examined the methylation status of the RTA promoter at the population level. We thus decided to further investigate the methylation status of this DNA fragment on individual viral genomes, using bisulfite genomic sequencing (BGS). Results showed that in latently infected S11E cells, majority of the CpG sites in the RTA promoter region were methylated (Fig. 2D, panel a). By contrast, analysis of the virion DNA or viral genome in *de novo* infected BHK-21 cells showed that most of the CpG sites in the RTA promoter were unmethylated (Fig. 2D, panels b and c). More importantly, after S11E cells were treated with 5 µM 5-AzaC for 24 hrs, the RTA promoter region was mostly demethylated (Fig. 2D, panel d). Taken together, these data indicated that distinctive methylation patterns were associated with MHV-68 at the RTA promoter during latency or lytic replication in cell culture. Furthermore, 5-AzaC treatment of S11E cells successfully induced demethylation of the RTA promoter region, however, demethylation only led to weak reactivation of MHV-68 from latency.

### 3. Trichostatin A (TSA) strongly reactivated MHV-68 from latency in S11E cells

**Figure 3:**
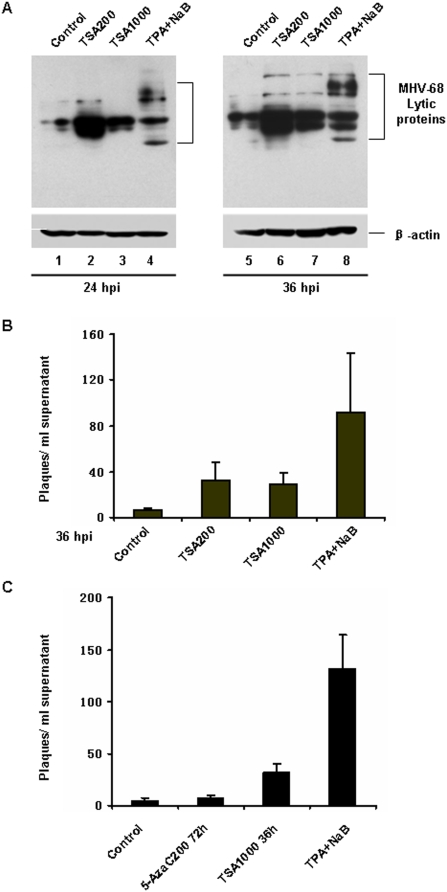
TSA strongly reactivated MHV-68 lytic replication from latency. (A) Detection of MHV-68 lytic protein by western blotting after S11E cells were induced by TSA. S11E cells were treated with 200 ng/ml or 1000 ng/ml TSA for 24 or 36 hrs, and then total cells were lysed and viral lytic proteins detected with anti- MHV-68 lytic antigens. TPA (25 ng/ml) plus NaB (4 mM) treatment served as a positive control. (B) Plaque assay on viral titer from the S11E cell supernatant after TSA treatment for 36 hrs. The experiments were repeated three times for each treatment. Standard deviations are expressed as error bars.

Our data above indicated that DNA demethylation is not sufficient for MHV-68 reactivation, at least in the S11E cells we tested. Since histone acetylation also plays a role in regulating gene transcription, we next investigated whether modification of histone acetylation of the MHV-68 genome could induce MHV-68 to go to lytic replication from latency in S11E cells. As shown in Fig. 3A, when S11E cells were treated with TSA for 24 or 36 hrs (200 ng/ml, lanes 2 and 6; or 1000 ng/ml, lanes 3 and 7), increased MHV-68 lytic protein expression was detected by western blotting using a polyclonal antibody against MHV-68 lytic antigens, indicating reactivation of MHV-68 virus. To quantitatively examine the effect of TSA induction, we measured virus titer present in the supernatant by plaque assay. After TSA induction for 36 hrs, virus titer increased considerably than that from mock treatment (Fig. 3B). These data demonstrated that TSA treatment successfully led to MHV-68 lytic replication in S11E cells. Because the reactivation efficiency in S11E cells may vary from time to time, we directly compared the effect of 5-AzaC and TSA on viral reactivation in the same experiment. Result shown in Fig. 3C confirmed that TSA induced MHV-68 reactivation more robustly than 5-AzaC did.

### 4. Histone acetylation modification at MHV-68 RTA promoter

**Figure 4:**
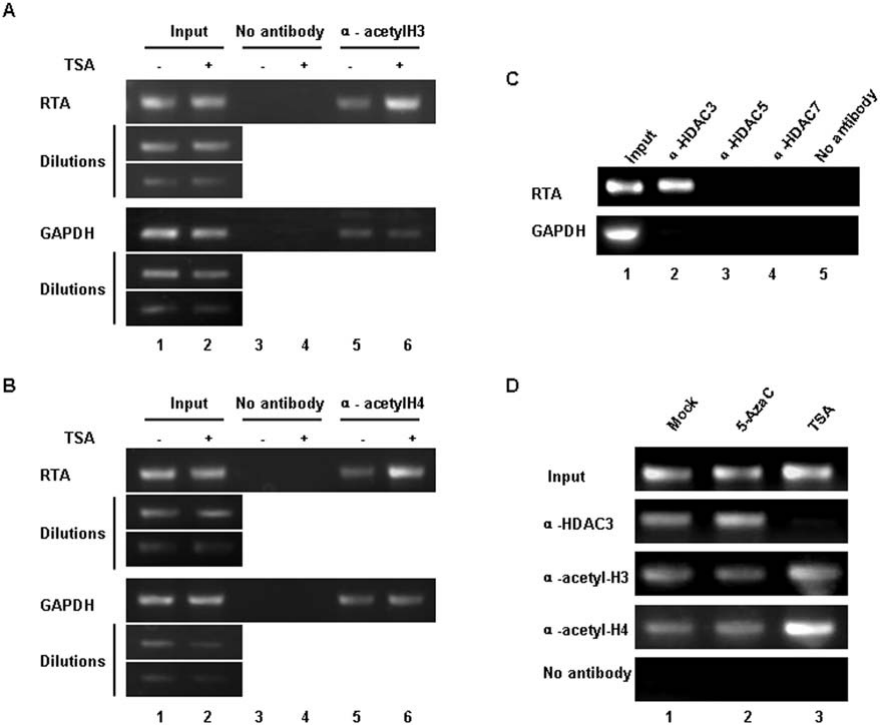
Histone acetylation modification at MHV-68 RTA promoter. (A) ChIP analysis of histone H3 acetylation level at MHV-68 RTA promoter. S11E cells were stimulated with TSA (200 ng/ml) for 4 hrs or mock-treated. Chromatin fragments were immunoprecipitated with an antibody against acetylated histones H3 (or no antibody control). Primers specific for RTA promoter or the GAPDH coding sequence were used to amplify the DNA isolated from the immunoprecipitated chromatin. The input DNAs and their dilutions were amplified to serve as positive controls. (B) ChIP analysis of histone H4 acetylation level at MHV-68 RTA promoter. Experiments were conducted as above, except an antibody against acetylated histone H4 was used. (C) HDAC3 was recruited to the MHV-68 RTA promoter. Recruitment of HDAC3, 5 or 7 to MHV-68 RTA promoter in S11E cells was investigated by ChIP. ChIP analysis of GAPDH coding sequence region is shown in the lower panel. Negative control (“No antibody”) and positive control (“Input”) were indicated. (D) ChIP analysis of HDAC3, acetylated histones H3 or acetylated histones H4 at RTA promoter. S11E cells were treated with 5-AzaC (10 µM, 36 hrs), TSA (200 ng/ml, 4 hrs) or left untreated, chromatin fragments immunoprecipitated and then analyzed by ChIP.

It has been reported that most MHV-68 viruses were present as episomes in S11E cells [25], therefore, the virus genomes are subject to chromatin modifications such as histone acetylation. We thus employed chromatin immunoprecipitation (ChIP) assay to examine whether histone acetylation modification occurred at the RTA promoter after TSA treatment. We first used an antibody against acetylated histone H3 to detect the change of histone H3 acetylation level at the RTA promoter. TSA treatment of S11E cells for 4 hrs resulted in up-regulation of histone H3 acetylation level, compared to that of mock treatment. As a control, TSA treatment did not change the level of histone H3 acetylation at GAPDH locus (Fig. 4A, lanes 5 and 6). Furthermore, the ChIP assay showed that the level of acetylated histone H4 at the RTA promoter also increased significantly after TSA treatment (Fig. 4B).

*In vivo*, histone acetylation and histone deacetylation are controlled by HATs and HDACs. Their balance is important for regulating gene expression. Since TSA is an inhibitor of HDACs, we next investigated whether TSA reactivation of MHV-68 was mediated through affecting HDAC binding at the RTA promoter. We used a panel of antibodies against individual HDACs in ChIP to investigate the recruitment of HDACs at the RTA promoter during latency. As shown in Fig. 4C, we found that HDAC3 was recruited to the RTA promoter in S11E cells (lane 2), however, HDAC5, 7, 1, 4, or 6 were not recruited (lanes 3 and 4, and data not shown).

We next examined whether association of HDAC3 with latent MHV-68 RTA promoter in S11E cells was indeed modulated by TSA treatment. As shown in Fig. 4D, treatment with TSA caused removal of most of the HDAC3 from the RTA promoter (lane 3). This is accompanied by increased acetylation level of histone H3 and histone H4, consistent with the results from Fig. 4A and 4B. As a comparison, treatment of S11E cells with 5-AzaC had little effect on HDAC3 binding, and the acetylation level of H3 and H4 at the RTA promoter remained unchanged compared to mock treatment (Fig. 4D, lanes 1 and 2). Taken together, these data indicated that HDAC3 plays an important role in suppressing RTA promoter transcription during viral latency.

### 5. Passive demethylation at the RTA promoter was associated with MHV-68 reactivation

**Figure 5:**
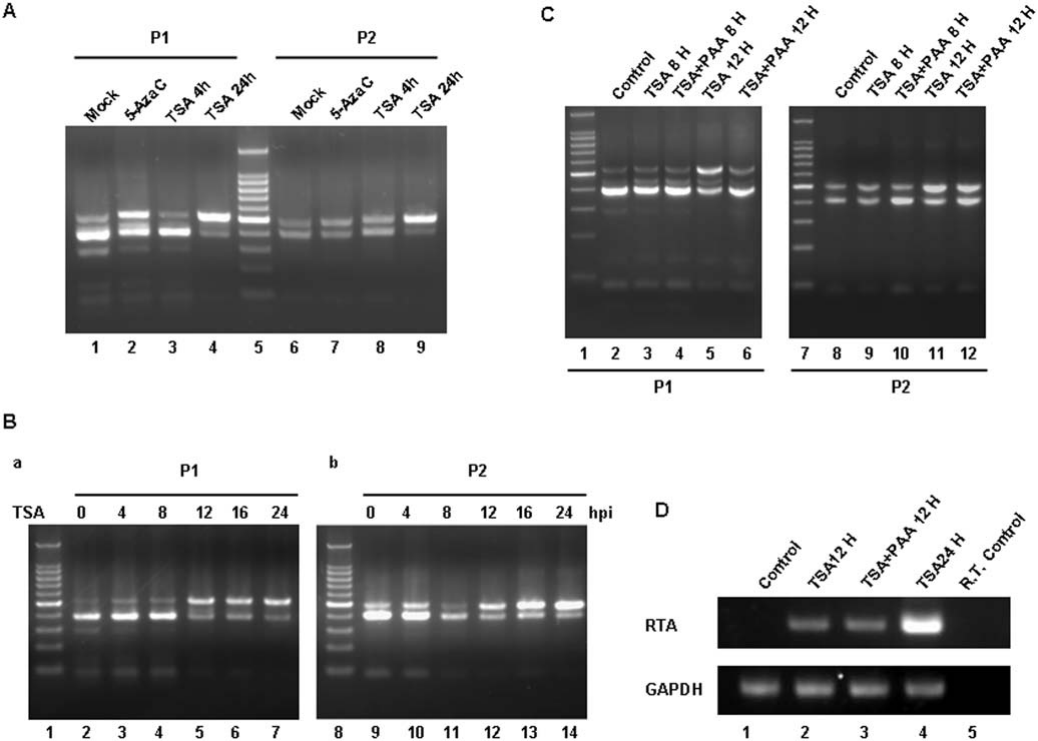
Passive demethylation at RTA promoter was associated with MHV-68 reactivation. (A) COBRA of methylation status at RTA P1 and P2 promoter. S11E cells were stimulated with 5-AzaC (10 µM, 24 hrs), TSA (200 ng/ml, 4 hrs), TSA (200 ng/ml, 24 hrs) or mock treated for COBRA. (B) Time course of methylation status change at the P1 and P2 fragments of RTA promoter in S11E cells. S11E cells were treated with TSA (200 ng/ml), and at 0, 4, 8, 12, 16 or 24 hrs post-induction, DNA was extracted for COBRA. (C) COBRA of methylation status at RTA promoter with PAA treatment. S11E cells were treated with TSA (200 ng/ml) and/or PAA (200 µg/ml) for 8 or 12 hrs, and DNA extracted for COBRA of the RTA P1 and P2 methylation status. (D) RT-PCR analysis of MHV-68 RTA mRNA expression after TSA plus PAA treatment. S11E cells were stimulated with TSA (200 ng/ml) 12 hrs, TSA (200 ng/ml) plus PAA (200 µg/ml) 12 hrs or TSA (200 ng/ml) for 24 hrs, and then total RNA isolated for RT-PCR. Analysis of GAPDH served as a control.

As described above, TSA treatment led to removal of HDAC3 complex from latent RTA promoter and viral reactivation (Fig. 4). The RTA promoter has distinctive methylation status during latency and lytic replication (Fig. 2). Therefore we asked whether TSA treatment could also result in change of the methylation status of RTA promoter. COBRA revealed that, after 4 hrs of TSA treatment, the RTA promoter methylation status was similar to that from mock treatment (Fig. 5A, lanes 1, 3, 6 and 8). However, after 24 hrs of TSA treatment, the RTA promoter region was mostly demethylated (Fig. 5A, lanes 4 and 9), and the extent of demethylation was more than that from 5-AzaC treatment for 24 hrs (Fig. 5A, lanes 2 and 7). A time course analysis of TSA treatment showed that the RTA promoter demethylation occurred between 8 to 12 hrs post-induction (Fig. 5B).

Two distinct demethylation mechanisms have been reported, active demethylation and passive demethylation. Passive DNA demethylation occurs through inhibition or under-maintenance of DNMTs throughout cycles of replication, while active DNA demethylation requires specific enzymatic reactions [26]. In order to investigate whether the RTA promoter demethylation we observed was caused by direct demethylation after TSA treatment or was a result of passive demethylation after viral genome replication, we applied phosphonoacetic acid (PAA), an inhibitor of DNA replication. As shown in Fig. 5C, after treatment with TSA alone for 12 hrs, the RTA promoter demethylation was observed as previously (lanes 5 and 11, compared to lanes 2 and 8). However, presence of PAA greatly reduced demethylation of the RTA promoter (lanes 6 and 12). Although it is possible that treatment with PAA may have an effect on virus gene transcription, such a possibility is not supported by the fact that RTA transcription level was not affected as detected by RT-PCR (Fig. 5D). Taken together, these data indicated that passive demethylation at the RTA promoter was associated with TSA induction of MHV-68 reactivation and most likely occurred on newly replicated viral genomes.

### 6. TSA and 5-AzaC did not act synergistically in S11E cells

**Figure 6:**
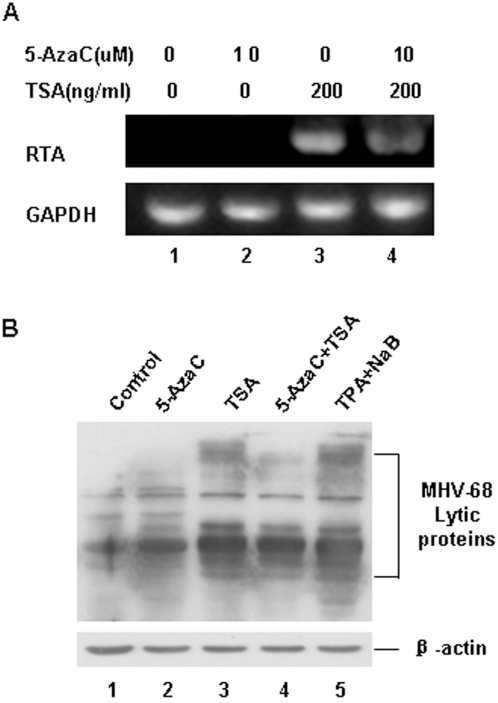
TSA and 5-AzaC did not act synergistically to induce MHV-68 reactivation. (A) RT-PCR analysis of MHV-68 RTA mRNA expression after 5-AzaC and/or TSA treatment. S11E cells were induced with 5-AzaC (10 µM) and/or TSA (200 ng/ml) for 24 hrs and then total RNA were isolated for RT-PCR, GAPDH mRNA was amplified as a control. (B) Western blotting analysis of MHV-68 lytic protein expression after 5-AzaC and/or TSA treatment. TPA (25 ng/ml) plus NaB (4 mM) treatment served as a positive control.

Our previous data suggested that 5-AzaC alone could reactivate MHV-68 very weakly, but TSA alone could reactivate MHV-68 strongly. We then went to determine whether these two chemicals could act synergistically to induce MHV-68 reactivation in S11E cells. Treatment of S11E cells with both 5-AzaC and TSA, when compared to TSA treatment alone, neither enhanced RTA transcription (Fig. 6A) nor increased MHV-68 lytic protein expression (Fig. 6B). These data indicated that 5-AzaC and TSA cannot act synergistically to reactivate RTA transcription in S11E cells.

### 7. Analysis of the methylation status and acetylation level of the RTA promoter *in vivo*

**Figure 7:**
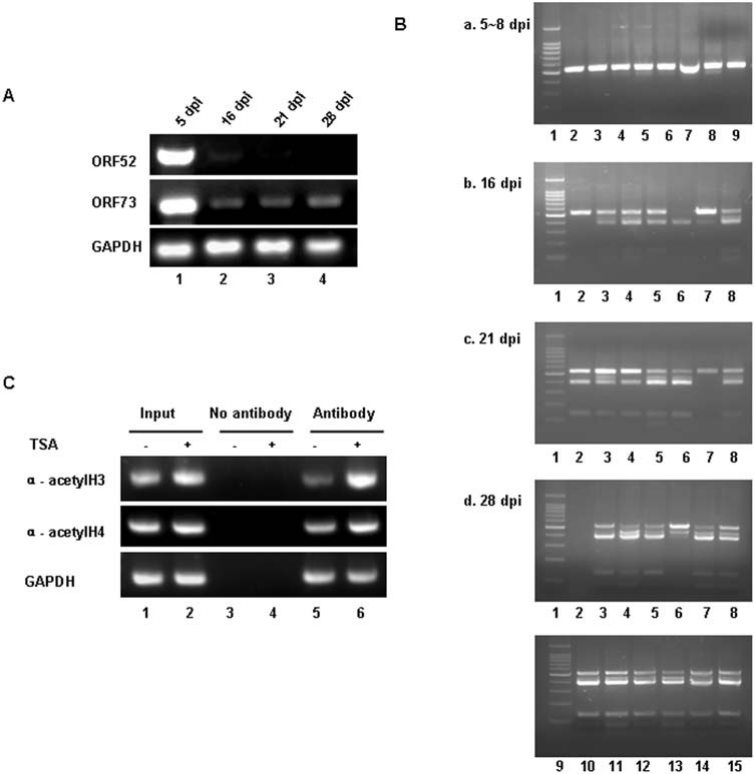
Analysis of methylation and acetylation status of the RTA promoter *in vivo*. (A) RT-PCR detection of ORF52 and ORF73 expression. Infected tissues were prepared mice at 5, 16, 21 and 28 dpi and mRNA extracted. The coding sequences for ORF52 and ORF73 were RT-PCR amplified and separated on a 2% agarose gel. (B) COBRA of methylation status of RTA promoter. DNAs were extracted from infected tissue at 5, 8, 16, 21 or 28 dpi respectively, bisulfite treated, amplified by PCR, digested with Taqα| and separated on a 2% agarose gel. (C) TSA treatment of MHV-68 latently infected mice. At 28 dpi, MHV-68 infected BALB/C mice were administrated with TSA by subcutaneous injection in the groin (2.4 mg/kg). Five hrs post-induction, ChIP assay were used to detect histones H3 and H4 acetylation level at the RTA promoter.

Compared to EBV and KSHV, MHV-68 is able to infect laboratory mouse, providing a useful model to study the basic biology of gammaherpesvirus infection *in vivo*. After intranasal (i.n.) infection, MHV-68 takes on acute virus replication in the lung, followed by B-cell-dependent spread of virus to the spleen and other lymphoid tissues. Latency is established predominantly in B cells, macrophages, dendritic and epithelial cells [27], [28], [29], [30]. To analyze the MHV-68 RTA promoter methylation status *in vivo*, we infected BALB/C mice by i.n. infection. At different days post infection (dpi), we performed RT-PCR analysis of viral gene expression to assess whether the virus was in lytic replication or latency. ORF52, encoding a viral tegument protein, is only expressed during lytic phase but not in latency, whereas the classic latency gene ORF73 is expressed during both phases. Consistent with previous report [27], [28], at 5 dpi, both ORF52 and ORF73 were abundantly expressed, indicating that MHV-68 was in lytic phase (Fig. 7A, lane 1). However, expression of ORF52 decreased and diminished at 16, 21 and 28 dpi, whereas ORF73 maintained a decreased level of expression even at 28 dpi (Fig. 7A, lanes 2–4), suggesting that MHV-68 had gone through the process of establishing to maintenance of latency. Concurrently, COBRA analysis showed that at 5 dpi, the RTA promoter region was nearly all unmethylated (Fig. 7B, panel a). As viral latency was established and maintained at 16, 21 and 28 dpi, increased level of methylation was observed at the RTA promoter (Fig. 7B, panels b–d). BGS of selected individual samples further confirmed the results (data not shown).

We also attempted to examine the acetylation level of the RTA promoter *in vivo*. As a comparison, we administered mice with latent MHV-68 infection (at 28 dpi) with TSA, since it has been reported that subcutaneous injection of TSA at 2.4 mg/kg did not give rise to obvious toxic effect on mice [31]. ChIP assay was performed to detect the acetylation level of histones H3 and H4 at the RTA promoter at 5 hrs post-treatment. The results showed that the acetylation level of histones H3 and H4 at the RTA promoter was low during viral latency *in vivo* (Fig. 7C, lane 5), and this level was up-regulated by TSA treatment (Fig. 7C, lane 6).

## Discussion

**Figure 8:**
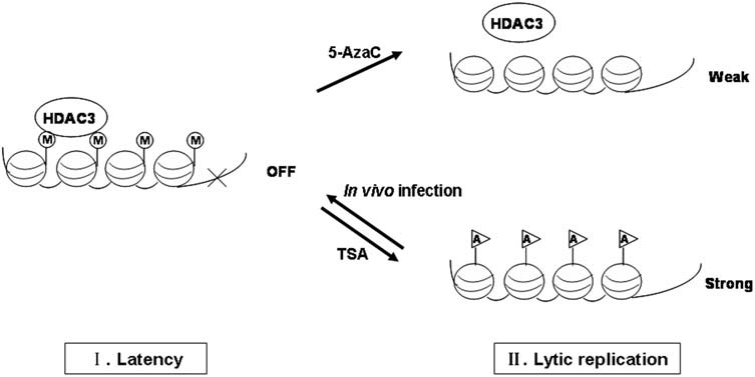
A working model for DNA demethylation and histone acetylation regulation of MHV-68. (A) In S11E cells or latent infection *in vivo*, the RTA promoter is methylated and HDAC3 complex is recruited to the RTA promoter to suppress transcription. (B) When treated with TSA, the HDAC3 complex is rapidly removed, the RTA promoter demethylated through a passive mechanism, and RTA transcription is turned on. (C) However, when treated with 5-AzaC, though the RTA promoter becomes demethylated, HDAC3 stays at the promoter and the RTA promoter remains suppressed. M: methylation; A: acetylation.

MHV-68 belongs to the gammaherpesvirus subfamily, which is characterized by common biological traits, latency and lytic replication. Previous studies have shown that both DNA methylation and histone acetylation regulate EBV and KSHV reactivation [18], [19], [20], [21], [22]. The role of epigenetic regulation in MHV-68 reactivation has not been reported. In this study, we investigated the effect of DNA methylation and histone acetylation associated with MHV-68 reactivation in S11E cells. The MHV-68 RTA promoter harbored distinctive methylation patterns during latency and lytic replication *in vitro* and *in vivo*. However, methylation inhibitor 5-AzaC was ineffective in reactivating MHV-68 to lytic phase. In contrast, HDAC inhibitor TSA strongly induced MHV-68 to go to lytic replication. During MHV-68 latency, HDAC3 complex was recruited to the RTA promoter to suppress transcription. TSA treatment led to removal of HDAC3 from the RTA promoter, up-regulation of histone H3 and H4 acetylation level, and viral reactivation. Reactivation is accompanied by passive demethylation at the RTA promoter. These findings led us to propose a model in which, at least in S11E cells, histone acetylation induced by TSA, but not DNA demethylation, is sufficient for effectively reactivating MHV-68 from latency (Fig. 8).

Many factors have been shown to be involved in MHV-68 reactivation. Firstly, several chemical reagents stimulate ORF50 gene expression and consequently lead to viral lytic reactivation [23], [24]. Additionally, some viral proteins, including RTA itself, and host cell signals activate RTA transcription and eventually lead to MHV-68 lytic replication [14] (unpublished data). Here, we show that chromatin remodeling also affects MHV-68 reactivation.

DNA methylation is an important mechanism for regulating gene expression. It has been well demonstrated that DNA methylation suppresses transcription, and transcription can be activated when treated with methylation inhibitors [32], [33]. Many tumor suppressor genes are repressed by hypermethylation, hence leading to cancer growth [34], [35]. In KSHV, the RTA promoter is methylated during latency[18]. The EBV genome is also highly methylated in latently infected cells, and the virus can be converted from latency to lytic replication by DNA methylation inhibitors such as 5-AzaC [21], [22], [36], [37]. Our study of MHV-68 showed that both *in vitro* and *in vivo*, the viral RTA promoter region was highly methylated during latency, whereas during *de novo* infection, *in vitro* or *in vivo*, the RTA promoter was mostly unmethylated (Fig. 2 and 7). Furthermore, treatment of S11E cells by 5-AzaC, although successfully resulted in demethylation of the RTA promoter (Fig. 2), is not sufficient to effectively induce MHV-68 reactivation (Fig. 1). Therefore, for MHV-68, it seems that methylation of the RTA promoter is tightly associated with establishment and maintenance of viral latency, however, demethylation of the RTA promoter alone is not sufficient to effectively reactivate the virus.

In a related study, Moser *et al* have shown that conditional deletion of DNMT 1 alleles from MHV-68 infected cells *in vivo* led to a severe ablation of viral latency [38]. DNMT 1 is a critical maintenance methyltransferase which maintains the DNA methylation patterns of the cellular genome during DNA replication. Since such a process is essential for the survival of the proliferating cells, the authors concluded that the proliferation of latently infected B cells is critical for the establishment of MHV-68 latency [38]. However, since the latent MHV-68 would have to replicate its genome in sync with the cellular genome during B cell proliferation, deletion of DNMT1 would also result in loss of methylation of the newly replicated viral genomes. Based on our study reported here, demethylation of the viral genome, especially of the RTA promoter, would lead to reactivation of MHV-68 into lytic replication and eventual lysis (and loss) of the latently infected cells, and hence may also be responsible for the severe ablation of the viral latency observed.

Many studies have reported that the activities of HATs and HDACs are linked to transcription [39], [40]. Up till now, 18 members of the HDACs family have been reported [41], [42], [43]. Based on size, catalytic domain, subcellular localization, and mechanism of deacetylation, HDACs are classified into four classes. Class I HDACs include HDAC1, 2 and 3, which have been showed to be sensitive to HDAC specific inhibitors such as TSA [44]. Previous data showed that TSA induced both KSHV and EBV reactivation [19], [20], [21], [45]. In KSHV, Lu *et al* reported that the RTA promoter is highly responsive to TSA as well as another HDAC inhibitor NaB. However, although the RTA promoter is found associated with several HDACs in latently infected cells, NaB treatment did not result in significant change in the association of HDACs. Instead, chromatin remodeling complex Snf5/Ini1 was recruited to the RTA promoter [19]. As for EBV, the scenarios are more complicated. Chang *et al* showed that TSA treatment of an EBV latently infected cell line P3HR1 resulted in acetylation of histone H4 at the BRLF1 promoter as well as transcription activation of the BRLF1, suggesting that histone acetylation at the RTA promoter induces EBV reactivation [20]. However, a more thorough analyses of the effect of several HDACs inhibitors (NaB, TSA and valproic acid) on multiple latent EBV cell lines by Countryman *et al* revealed that those HDAC inhibitors, though capable of promoting hyperacetylation at the promoters for BZLF1 (ZEBRA) and BRLF1 (RTA), only induced EBV go to lytic cycle in some cell line background [21]. Thus, open chromatin at EBV BZLF1 and BRLF1 promoters by itself is not sufficient to activate EBV lytic cycle gene expression [21]. In MHV-68, our data showed that HDAC3 was recruited to the RTA promoter in latently infected S11E cells to suppress RTA transcription, and TSA treatment reactivated MHV-68 through dissociation of HDAC3 from the RTA promoter, accompanied by up-regulation of histone H3 and histone H4 acetylation levels (Fig. 3 and 4).

Only HDACs residing in the nucleus can deacetylate histones and repress transcription, and proteosome degradation appears to be a mechanism of regulation of HDAC function [46], [47], [48], [49]. We thus examined the distribution and integrity of HDAC3 after TSA treatment of S11E cells. Consistent with previous finding that HDAC3 can be found in both the nucleus and cytoplasm [50], [51], our western blot analysis also detected HDAC3 in both fractions (data not shown). However, HDAC3 was neither translocated nor degraded after TSA treatment (data not shown). Therefore, the exact mechanism governing the role of HDAC3 in regulating MHV-68 reactivation remains to be further investigated.

Nonetheless, it should be noted that HDACs generally exist as a component of stable large multi-subunit complexes, and the activities of most if not all HDACs are regulated by protein-protein interactions [52], [53], [54]. There are also cross-talks between the epigenetic processes of DNA demethylation and histone modifications [55], [56]. It has been well demonstrated that DNA methylation can repress transcription through several mechanisms, e.g. directly inhibiting binding of transcription factor to DNA, and indirectly through the effects of methyl-CpG binding proteins. Thus, methyl-CpG binding proteins (e.g. MeCP2 and MBDs) can be recruited to methylated CpG where they can act as mediators of transcriptional repression through the association with HDAC containing repressor complexes [57]. For instance, HDAC1 and HDAC2 exist in NuRD (Nucleosomes remodeling and deacetylase) as well as at least two other distinct complexes to repress transcription, and the NuRD complex also contains MBD3 [58], [59]. Therefore, demethylation will result in removal of MeCP or MBDs, leading to dissociation of the NuRD complex containing HDAC1/HDAC2 and hence activating gene transcription. Consistently, previous reports showed that in KSHV, it was HDAC1, but not the HDAC3, which was recruited to the RTA promoter [19], and demethylation of the KSHV ORF50 promoter strongly induced viral reactivation from latently infected cell line [18].

In contrast, HDAC3 depends upon SMRT (Silencing mediator of retinoid and thyroid receptor) and N-CoR (Nuclear receptor co-repressor) complexes to remodel the promoter structure and repress transcription [52], [60], [61], [62]. Till now, it is regarded that HDAC3 does not interact with MBDs [57], [59]. Consistently, our data showed that, during MHV-68 latency, HDAC3 was recruited to the RTA promoter (Fig. 4C), however, demethylation of the RTA promoter could not remove the HDAC3 complex (Fig. 4D) and thus was not sufficient to effectively reactivate the virus from latency. This may also offers an explanation for why we could not detect synergistic effects between 5-AzaC and TSA treatment for induction of MHV-68 reactivation (Fig. 6), in contrary to many reports showing that inhibiting DNA methylation and histone deacetylation can synergistically induce gene expression [63], [64], [65].

To our knowledge, this study is the first to investigate the DNA demethylation and histone acetylation associated with gammaherpesvirus reactivation *in vitro* and *in vivo* in the same system. In this system we found that histone acetylation plays a much more significant role in regulating MHV-68 reactivation. As a comparison, KSHV can be reactivated from latently infected BCBL-1 cells by either the DNMTs inhibitor reagent such as 5-AzaC [18], or the HDAC inhibitors such as NaB or TSA [19]. These data show that both reagents were strong to reactivate the virus, though the reactivation efficiencies could not be directly compared due to the usage of different investigating methods. We hypothesize that in the MHV-68 system, DNA methylation is critical for maintaining the viral latency status, while histone acetylation is important for chromatin structure remodeling at the RTA gene promoter and accordingly induces MHV-68 reactivation. It should be noted that, till now only one naturally occurring MHV-68 latently infected cell line has been isolated. Performing similar analysis in additional MHV-68 latently infected cell lines (once available) will yield more information and shed more light on the role of epigenetic modification in controlling MHV-68 reactivation.

## Materials and Methods

### 1. Cell culture and chemical treatment

S11E is a clonal cell line of S11, which was established from a B-cell lymphoma developed in an MHV-68 infected mouse and contains latent MHV-68 [25]. S11E cells were cultured in RPMI 1640 medium containing 15% FBS, penicillin (100 U/ml), streptomycin (100 mg/ml) and 50 µM β-mercaptoethanol. 293T and BHK-21 were maintained in Dulbecco's modified Eagle's Medium (DMEM) plus 10% FBS, penicillin (100 U/ml) and streptomycin (100 mg/ml). 5-azacytidine (5-AzaC), trichostatin A (TSA), 12-O-tetradecanoylphorbol-13-acetate (TPA) and sodium butyrate (NaB) were purchased from Sigma. These chemicals were used to induce S11E cells at various concentrations for various times in different experiments. For cell treatment with 5-AzaC for a total of 36, 48 or 72 hrs, media with freshly added 5-AzaC was replaced at 24-hr interval. Phosphonoacetic acid (PAA) was also purchased from Sigma, and S11E cells were treated at a concentration of 200 µg/ml.

### 2. Mouse administration and *in vivo* infection

Four to six week-old, specific pathogen free (SPF) female BALB/C mice were purchased from Beijing Laboratory Animal Research Center. Maintenance of mice and experimental procedures were approved by the Animal Welfare and Research Ethics Committee of the Institute of Biophysics, Chinese Academy of Sciences. For *in vivo* infection, the mice were anesthetized intraperitoneally (i.p.) with pentobarbital sodium (50 mg/kg body weight), and then inoculated intranasally ( i.n.) with 20 µl of viral stock contain 4.0×10^5^ PFU of MHV-68 viruses. Control mice were inoculated i.n. with 20 µl of phosphate-buffered saline (PBS). For TSA induction experiment, the mice were injected directly by subcutaneous route in the groin at a dosage of 2.4 mg/kg.

### 3. Western blotting

Total cell extracts were prepared using standard protocols and resolved on a SDS-polyacrylamide gel. After transferring to polyvinylidene fluoride (PVDF) membrane (Millipore) and blocking with 5% milk, the MHV-68 lytic proteins were detected using a rabbit polyclonal antibody (a kind gift from Prof. Ren Sun, UCLA) at 1∶2000 dilution, followed by HRP-conjugated anti rabbit IgG (Beijing Zhongshan Golden Bridge Biotechnology Co., LTD) at 1∶10,000 dilution. β-actin was detected by a mouse monoclonal antibody (Sigma) at 1∶5000 dilution, followed by HRP-conjugated anti mouse IgG (Beijing Zhongshan Golden Bridge Biotechnology Co., LTD) at 1∶10,000 dilution. Protein bands were visualized by the chemiluminescence detection system (Millipore).

### 4. Plaque assay

BHK-21 cells were grown in 12- well plates to 20–30% confluency. Cells were infected in duplicates with serial 10-fold dilutions of cell culture supernatants, and incubated at 37°C for 1 hr, with gentle rocking every 15 min. After that the inoculum was aspirated out, cells were then overlaid with 2 ml of DMEM containing 10% FBS, 1% antibiotics and 1% methyl cellulose agar (Sigma). After 5 days of incubation, the plaques were stained and counted under a microscope to determine viral titers.

### 5. Genomic DNA Isolation, Q-MSP, COBRA and BGS

Genomic DNA was isolated by standard phenol-chloroform extraction procedure. In brief, S11E cells were washed twice with ice-cold phosphate-buffered saline (PBS), and then incubated at 55°C overnight with digestion buffer (100 mM NaCl, 25 mM EDTA, 10 mM Tris-HCl [pH 8.0], 0.5% sodium dodecyl sulfate, 0.1 mg/ml proteinase K). DNA was extracted twice with phenol-chloroform-isoamyl alcohol (25∶24∶1), and collected by ethanol precipitation.

Bisulfite treatment of genomic DNA was preformed in agarose by a special method described previously [66], [67]. In brief, 700 ng DNA was digested by Bgl||, which does not cut within the region of interest, and denatured in 0.3 M NaOH at 50°C for 15 min. The denatured DNA was mixed with melted 2% (w/v) LMP agarose (Amresco) to form beads, and incubated with 2.5 M sodium bisulfite (Sigma) at pH 5.0 and 0.5 mM hydroquinone (Sigma) at 50°C for 4 hrs. The beads were washed by TE to remove agarose, incubated with 0.2 M NaOH for 30 min for desulfonation, and washed again by TE to remove NaOH solution. The beads could then used for PCR directly.

The 1 kb MHV-68 RTA promoter region was divided into two fragments for convenience of analysis, defined as P1 and P2, which include a total of 15 CpG sites. For quantitative methylation-specific PCR (Q-MSP) assay, beads were amplified by Bio-Rad MyiQ single-color real-time PCR detection system with methylated primers or unmethylated primers respectively, and the results were quantified based on standard reactions performed at the same time. For Bisulfite Sequencing Analysis (BGS), beads were amplified by semi-nested PCR, and PCR products recovered and cloned into pMD18-T vector (TaKaRa) for sequencing. For Combined bisulfite restriction analysis (COBRA), beads were also amplified by semi-nested PCR and DNA recovered, followed by digestion with Taqα| (NEB) for 2 hrs, and visualized by ethidium bromide staining of 2% agarose gels. All PCR primers are listed in Table 1.

**Table 1:** Primers used in this study.

Primers	DNA Sequences (5′ - 3′)	Coordinates on MHV-68 or Cellular Genome
**Primers for BGS**		
P1-Forward	GGTTTTTGTGTAGAATTTTTGATTATGA	NC_001826, 65695-65722
P1-Reverse	CCAACCTCACCAACTTTTACAATA	NC_001826, 66235-66258
P1-Nest	AGTTATATTTTGTATATAAATATTTATGGT	NC_001826, 65728-65757
P2-Forward	TTTTTTGAATAGAGTGAGAAGGGTAG	NC_001826, 66355-66380
P2-Reverse	TCAAACTAATAACAACACTTTAATTTTTAA	NC_001826, 66858-66879
P2-Nest	TAGGTATATAATAAAATTTTTTGGAATT	NC_001826, 66378-66405
**Primers for MSP**		
P1-M-Forward	TGTTGGTTACGTTTAGGTATTCGA	NC_001826, 65791-65814
P1-M-Reverse	ATCTCACTAAAAACACTCCAACGAC	NC_001826, 66060-66084
P1-U-Forward	GTTGGTTATGTTTAGGTATTTGA	NC_001826, 65792-65814
P1-U-Reverse	ATCTCACTAAAAACACTCCAACAAC	NC_001826, 66060-66084
P2-M-Forward	GTATTACGAGGGAATTTTTGTAGC	NC_001826, 66753-66776
P2-M-Reverse	ATTTTTAATAAAATACTAATCTATCTACGT	NC_001826, 66828-66857
P2-U-Forward	TATTATGAGGGAATTTTTGTAGTGA	NC_001826, 66736-66760
P2-U-Reverse	ATTTTTAATAAAATACTAATCTATCTACAT	NC_001826, 66828-66857
**Primers for ChIP**		
RTA-Forward	CTCTGTCAGATGTGACCATGAG	NC_001826, 66501- 66522
RTA-Reverse	AAAATGTTTACCTACCTTATCGGCTG	NC_001826, 66785-66810
GAPDH-Forward	CACCCAGAAGACTGTGGATG	M_001001303, 601-620
GAPDH-Reverse	CGAAGGTGGAAGAGTGGGAG	M_001001303, 919-938
**Primers for RT-PCR**		
RTA-Forward	CTACATACCTACTCCCAACTCAG	NC_001826, 68782-68804
RTA-Reverse	ATTTACCTCCTCATCGCTCT	NC_001826, 68892-68911
ORF52-Forward	AGGAATTCGGTCAGGCGCTGTCTCATCAGA	NC_001826, 71056-71075
ORF52-Reverse	TCGGTACCTTATTCATGATCATGTCTGTGTC	NC_001826, 71342-71364
ORF73-Forward	TCCCTGGCTGGACTCCTCAT	NC_001826, 104692-104711
ORF73-Reverse	CCCACCGACTACACGCAACA	NC_001826, 104838-104857
GAPDH- Forward	TGAAGCAGGCATCTGAGGG	M_001001303, 837-855
GAPDH-Reverse	CGAAGGTGGAAGAGTGGGAG	M_001001303, 919-938

### 6. ChIP assay

Chromatin immunoprecipitation (ChIP) protocol was performed according to Upstate Company online protocol and a procedure described previously [68]. In brief, S11E cells were treated with or without TSA (200 ng/ml) for 4 hrs or 5-AzaC (10 µM) for 36 hrs (with fresh media every 24 hrs), then cross-linked with formaldehyde to a final concentration of 1% for 10 min at 37°C. The cells were washed in ice-cold PBS twice, resuspended in sodium dodecyl sulfate (SDS) lysis buffer and incubated for 20 min on ice. Lysates were sonicated to produce DNA fragments of an average length at 300–1000 bp. Extracts were then diluted 10-fold with immunoprecipitation (IP) dilution buffer. Two hundred microliters of the diluted sample (10%) was used as input controls and 1.8 ml of diluted sonicated extract for IP. After pre-clearing with Protein G Agarose for 30 min at 4°Cwith agitation, appropriate antibodies (anti-acetyl-histone H3 [Upstate], anti-acetyl-histone H4 [Upstate], anti-HDAC1, 3, 4, 5, 6, 7 [Cell signaling kit], anti-HDAC3 [Abcam]) were incubated overnight at 4°C with rotation. To collect the immune complexes, appropriate Protein G Agarose mixture was added to each reaction mixture and the mixture was rotated for 2 hrs at 4°C. Beads were centrifuged and washed for 5 min at 4°C with each of the following: low salt, high salt, LiCl, and Tris-EDTA buffer. The immune complexes were eluted by incubation in elution buffer, and supernatants were isolated and further incubated for 4 hrs at 65°C to reverse cross-linking. Input controls were treated in the same manner at this point. After reverse cross-linking, proteinase K was added and the mixture was incubated for 1 hr at 45°C. DNA was deproteinized by phenol-chloroform extraction and ethanol precipitation in the presence of 20 µg of glycogen. DNA was washed in 70% ethanol, dried, and resuspended in 20 µl of TE. For a typical PCR, 2 to 5 µl of the 20 µl total DNA was amplified for 22 to 34 cycles and visualized by ethidium bromide staining of agarose gels. Primers for RTA promoter and glyceraldehyde-3-phosphate dehydrogenase (GAPDH) coding sequence are listed in Table 1.

### 7. RNA isolation and RT-PCR

Total cellular RNA was extracted with TRIzol reagent according to the recommendations of the supplier (Invitrogen) and quantified using GeneQuant pro (Amersham Biosciences). Two microgram of RNA was reverse transcribed by moloney murine leukemia virus reverse transcriptase (M-MLV) using Oligod (T) 15 primer (TaKaRa). RTA and the constitutively expressed housekeeping gene GAPDH coding sequence were amplified by PCR using RT-PCR primers (see Table 1). PCR products were resolved by 2% agarose gel electrophoresis and visualized by ethidium bromide staining.
